# Needed Sleep

**Published:** 1892-04

**Authors:** 


### NEEDED SLEEP.

That the amount of sleep required by different individuals is decidedly different has almost passed into an axiom. Persons who are very energetic naturally require a great deal of sleep, and children and young people who are growing require at least nine or ten hours of sleep. Invalids or people advanced in life should sleep as long as they can, as there is no restorer of tired nature like sleep. To get a refreshing sleep the brain must cease to act. It would be curious to trace how many cases of irritability, or of functional diseases of the nerves, are due to lack of proper sleep. Little children should literally go to bed with the chickens. They should have an early supper, at half-past five, and be put to bed directly after. This should be kept up till the child is seven or eight years old, when the bed time hour may be changed from 5 o'clock till 7. A growing girl should certainly go to bed as early as 8 o'clock. The old Norman law which commanded that all fires should be covered and lights put out at the ringing of the curfew bell, though looked upon as a tyrannical measure, was, from a hygienic point of view, a wise one. Considerable harm has been done by arbitrary rules in the matter of sleep. The fact that Napoleon was able to exist with six hours' sleep, if it were true, proves nothing but his exceptional endurance. It is said that general Grant once said that he could do nothing without nine hours' sleep.

There has been considerable discussion as to what is the best position in sleep. Most physicians will tell you that you should lie on the right side, but no definite directions can be given. A weakness of the lungs may cause the sleeper to rest more comfortably on the left side. Again in depressing illness, the patient usually lies flat on his back, and this position seems, in general, to contribute the greatest amount of rest to the muscles, yet few people would find it a comfortable one. A position which has been advocated with considerable show of reason, is that of lying partly on the face. Probably no healthful person sleeps altogether in either one of them, but varies his position during his resting hours.

The best bed coverings are light woolen blankets. The impervious cotton comfortables so much used are the most unwholesome of any covering. A hair mattress is conceded now to be the very best bed, and a good hair bolster is the most wholesome head rest. Sleeping with a number of pillows under the head is certainly injurious, as it tends to raise the head into a cramped unnatural position. The fashion of double beds is one greatly to be deprecated, and two single beds placed side by side are taking their place in many cases. So high an authority as "The London Lancet" says, in discussing the question: "Nothing will so derange the nervous system of a person who is eliminative in nervous force as to lie all night in bed with another who is absorbent of nervous force. The latter will sleep soundly all night and arise refreshed in the morning, while the former will toss restlessly, and awake in the morning fretful, peevish, fainthearted and discouraged. No two persons, no matter who they are, should habitually sleep together.

